# Protective Effects of Extracts and Flavonoids Isolated from *Scutia buxifolia* Reissek against Chromosome Damage in Human Lymphocytes Exposed to Hydrogen Peroxide

**DOI:** 10.3390/molecules17055757

**Published:** 2012-05-14

**Authors:** Aline Augusti Boligon, Michele Rorato Sagrillo, Luiz Filipe Machado, Olmiro de Souza Filho, Michel Mansur Machado, Ivana Beatrice Manica da Cruz, Margareth Linde Athayde

**Affiliations:** 1 Program of Post-Graduation in Pharmaceutical Sciences, Federal University of Santa Maria, Campus Camobi, Santa Maria, RS, 97105-900, Brazil; 2 Franciscan University Center – UNIFRA, Andradas Street, 1614, Santa Maria, RS 97010-032, Brazil; 3 Program of Post-Graduation in Pharmacology, Federal University of Santa Maria, Campus Camobi, Santa Maria, RS, 97105-900, Brazil; 4 Federal University of Pampa – UNIPAMPA, Uruguaiana, RS 97500-970, Brazil

**Keywords:** *Scutia buxifolia*, flavonols, lymphocyte culture, chromosomes damage, HPLC/DAD

## Abstract

Flavonoids are claimed to protect against cardiovascular disease, certain forms of cancer and ageing, possibly by preventing initial DNA damage. Therefore, we investigated the protective effects of crude extract, ethyl acetate fraction and flavonoids (quercetin, quercitrin, isoquercitrin and rutin) isolated from the leaves from *Scutia buxifolia* against chromosome damage induced by H_2_O_2 _in human lymphocytes by analyzing cellular growth rate, cell viability, mitotic index and chromosomal instability. We found a differential response among the compounds tested, with the ethyl acetate fraction being more effective than the crude extract, a difference perhaps related to the presence of the antioxidants identified and quantified by HPLC/DAD. In general, quercetin, isoquercitrin and rutin recovered the mitotic index and chromosomal instability more than quercitrin after treatment with hydrogen peroxide.

## 1. Introduction

In recent years, focus on plant research has increased all over the World [1]. The consumption of plant products is associated with a lowering risk of number of chronic diseases, including atherosclerosis and cancer [2]. These beneficial effects have been partly attributed to antioxidants, which may play important roles in inhibition of free radicals and oxidative chain-reactions within tissues and membranes [3].

Phenolic compounds, such as flavonoids, phenolic acids, coumarins and tannins are secondary plant metabolites, and an important part of both human and animal diets [4,5]. They exert a wide variety of biological effects, including anticarcinogenic, antimutagenic and antioxidative activities [6,7,8,9]. The antioxidant activity of these compounds is mainly due to their redox properties, which allow them to act as reducing agents or hydrogen-atom donors [10]. Additionally, phenolics and flavonoids are known to possess antimutagenic activity, among other important biological properties [11].

*Scutia buxifolia *Reissek belongs to the Rhamnaceae family and is popularly known as “coronilha”. It is native tree from South America, with a dispersion area that comprises Rio Grande do Sul State in Brazil, and the countries of Argentina and Uruguay. The root bark infusion is popularly used as a cardiotonic, antihypertensive and diuretic [12]. Antimicrobial activities of some cyclopeptide alkaloids isolated from the root bark of this species were reported by Morel *et al.* [13] who used the bioautography method.

Extracts from leaves, twigs and stem bark of *S. buxifolia* were considered toxic after an *Artemia salina* assay, and in addition showed *in vitro* antimicrobial and antimycobacterial activity against a panel of microorganism strains [14,15]. Extracts from the leaves and stem bark of *S.* *buxifolia *were effective inhibitors of thiobarbituric acid reactive species (TBARS) production and also presented 1,1-diphenyl-2-picrylhydrazyl (DPPH) radical scavenger activity. Quercetin, quercitrin, isoquercitrin and rutin were isolated from *S. buxifolia*, indicating that this plant contains promising compounds to be tested as potential drugs for the treatment of diseases resulting from oxidative stress [16]. In line of these findings, this study compares the ability of the crude extract, ethyl acetate fraction and four flavonoids (quercetin, rutin, isoquercitrin and quercitrin) isolated of the leaves from *S. buxifolia *to modulate the DNA damage induced in lymphocytes by hydrogen peroxide.

## 2. Results

### 2.1. HPLC Analysis

**Figure 1:**
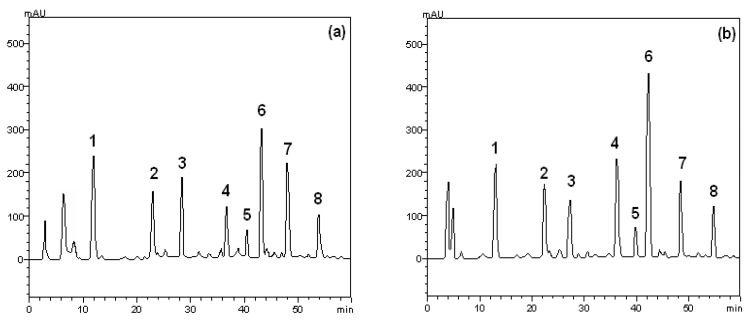
High performance liquid chromatography profile of crude extract (**a**) and ethyl acetate fraction (**b**) from the leaves of *S. buxifolia*. Gallic acid (peak 1), chlorogenic acid (peak 2), caffeic acid (peak 3), rutin (peak 4), isoquercitrin (peak 5), quercitrin (peak 6), quercetin (peak 7) and kaempferol (peak 8). Chromatographic conditions are described in the material and methods section.

The HPLC profile of crude extract and ethyl acetate from the leaves of *S. buxifolia* was also acquired (Figure 1). Leaves of *S. buxifolia* contains other minor compounds in addition to gallic acid (retention time-t_R_ 12.4 min, peak 1), chlorogenic acid (t_R_ = 23.1 min, peak 2), caffeic acid (t_R_ = 27.6 min, peak 3), rutin (t_R_ = 37.5 min, peak 4), isoquercitrin (t_R_ = 40.1 min, peak 5), quercitrin (t_R_ = 43.4 min, peak 6), quercetin (t_R_ = 48.2 min, peak 7) and kaempferol (t_R_ = 54.9 min, peak 8).

Since extracts of natural origin usually contain a range of chemically diverse constituents occurring in varying concentrations, it is important to use chromatographic methods to analyze these inherently complex mixtures. The HPLC profile of crude extract and ethyl acetate fraction was acquired, as well the quantification of quercetin, rutin, kaempferol, and gallic, chlorogenic and caffeic acids by HPLC-DAD based on references standards calibration curves (Table 1).

**Table 1:** Phenolics and flavonoids composition of *Scutia buxifolia* leaves; LOD and LOQ variations for compounds.

Compounds		Crude extract		Ethyl acetate fraction		LOD (μg/mL)	LOQ (μg/mL)
		Quantities (mg/g)		Quantities (mg/g)			
Gallic acid		18.1 ± 0.09 ^a^		34.6 ± 0.10 ^b^		0.041	0.125
Chlorogenic acid		4.9 ± 0.16 ^a^		22.1 ± 0.07 ^b^		0.030	0.992
Caffeic acid		7.3 ± 0.04 ^a^		19.8 ± 0.13 ^b^		0.026	0.079
Rutin		14.2 ± 0.05 ^a^		48.1 ± 0.18 ^b^		0.028	0.084
Isoquercitrin *		1.5 ± 0.07 ^a^		6.6 ± 0.04 ^b^		-	-
Quercitrin *		26.5 ± 0.03 ^a^		183.2 ± 0.05 ^b^		-	-
Quercetin		42.1 ± 0.11 ^a^		27.1 ± 0.03 ^b^		0.033	0.100
Kaempferol		5.6 ± 0.02 ^a^		15.5 ± 0.17 ^b^		0.021	0.063

Results are expressed as mean ± standard deviation (SD) of three determinations. Different letters in each column represent significant differences using analysis of variance followed by Tukey test (*p* values < 0.005 were considered as significant). * Quantified as quercetin. LOD: limit of detection; LOQ: limit of quantification.

### 2.2. Cell Viability

**Figure 2:**
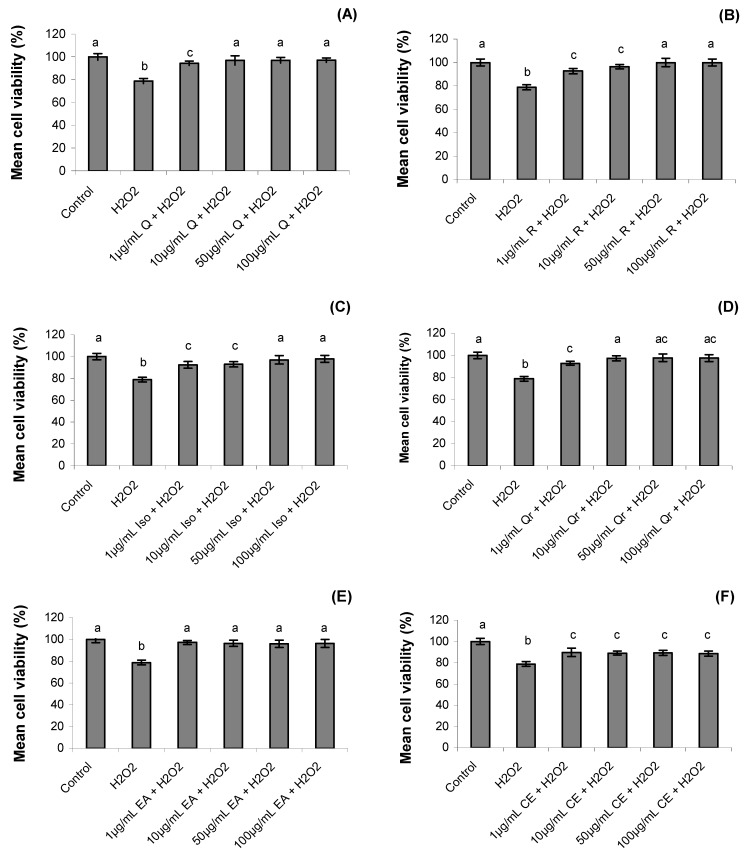
Effect of quercetin (Q), rutin (R), isoquercitrin (Iso), quecitrin (Qr), ethyl acetate fraction (EA) and crude extract (CE) of *S. buxifolia* on human lymphocyte cell viability incubation with H_2_O_2_. Concentration range of quercetin (**A**), rutin (**B**), isoquercitrin (**C**), quercitrin (**D**), ethyl acetate fraction (**E**) and crude extract (**F**). Results are mean ± SD for *n = *3 (*p = *0.0001). Different letters in each treatment represent significant differences using analysis of variance followed by Tukey test, *p* = 0.0001.

After one hour the cell viability decreased significantly in hydrogen peroxide treatment (78 ± 2.2%) when compared to control treatment (100 ± 2.9%) (*p *= 0.0001). Treatments with *S. buxifolia* crude extract, ethyl acetate fraction and isolated compounds (rutin, quercetin, isoquercitrin and quercitrin) increased the cell viability, despite the presence of hydrogen peroxide (*p *= 0.001). Rutin treatments at 50 µg/mL and 100 µg/mL concentrations reverted 100% of mortality associated to hydrogen peroxide (Figure 2).

### 2.3. Mitotic Index

**Figure 3:**
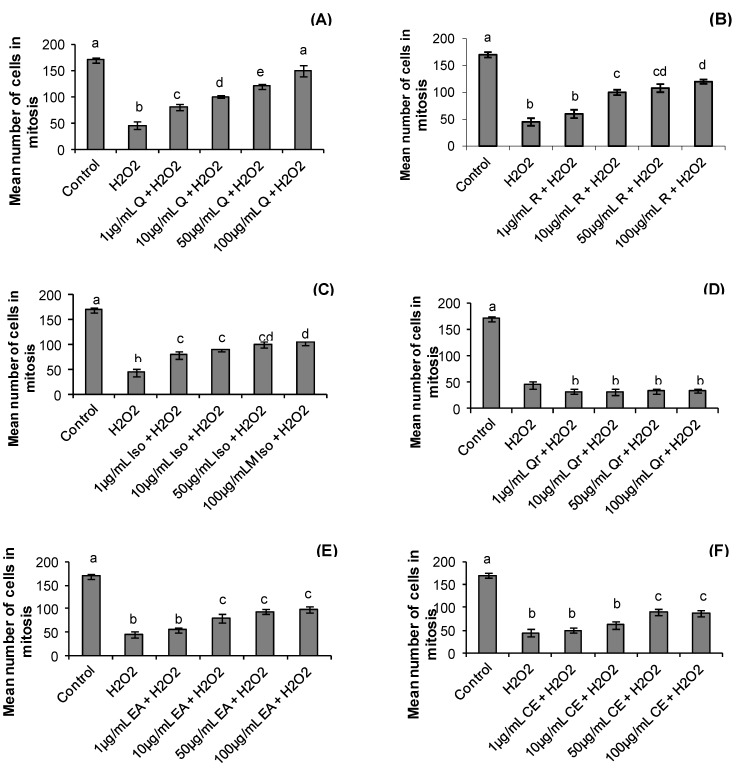
Effect of quercetin (Q), rutin (R), isoquercitrin (Iso), quecitrin (Qr), ethyl acetate fraction (EA) and crude extract (CE) of *S. buxifolia* on human lymphocyte number of cells in mitosis incubation with H_2_O_2_. Concentration range of quercetin (**A**), rutin (**B**), isoquercitrin (**C**), quercitrin (**D**), ethyl acetate fraction (**E**) and crude extract (**F**). Results are mean ± SD for *n = *3 (*p = *0.0001). Different letters in each treatment represent significant differences using analysis of variance followed by Tukey test, *p* = 0.0001.

Although the treatment period with hydrogen peroxide and *S. buxifolia* compounds was short (one hour) we observed that the treatments affected the mitotic index. The mean number of metaphases was higher in control group when compared with all treatments (*p* = 0.001). On the other hand, the hydrogen peroxide and all treatments with quercitrin presented lower number of metaphases; the other treatments only at low concentrations had no effect on number of cells in mitosis (Figure 3).

### 2.4. Chromosomal Instability

**Figure 4:**
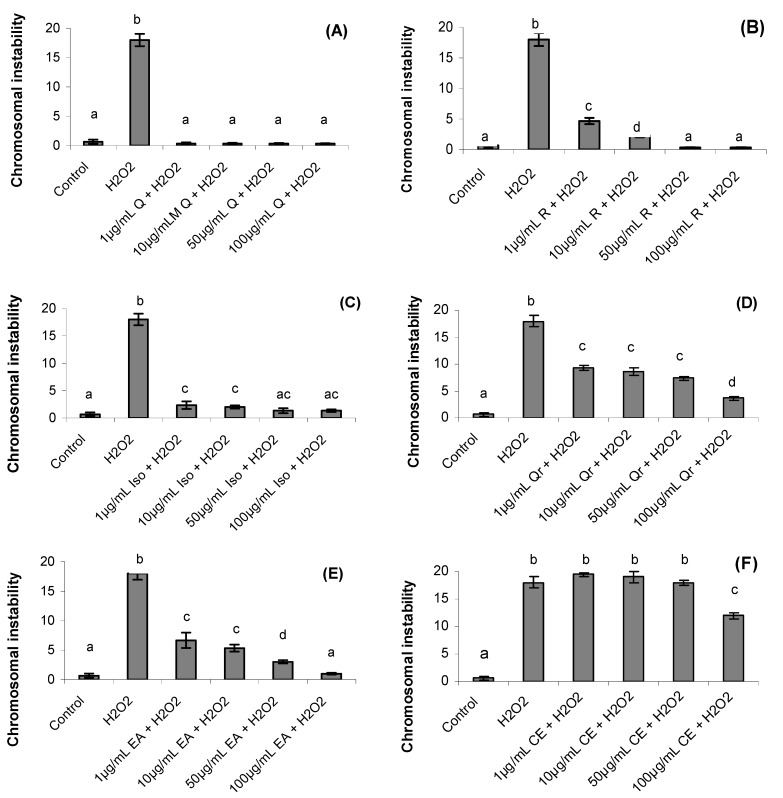
Effect of quercetin (Q), rutin (R), isoquercitrin (Iso), quecitrin (Qr), ethyl acetate fraction (EA) and crude extract (CE) of *S. buxifolia* on chromosomal instability incubation with H_2_O_2_. Concentration range of quercetin (**A**), rutin (**B**), isoquercitrin (**C**), quercitrin (**D**), ethyl acetate fraction (**E**) and crude extract (**F**). Results are mean ± SD for *n =* 3 (*p = *0.0001). Different letters in each treatment represent significant differences using analysis of variance followed by Tukey test, *p* = 0.0001.

The chromosomal instability is shown in Figure 4. After one hour the chromosomal instability increased significantly in hydrogen peroxide treatment (18 ± 1.05) when compared to control treatment (0.66 ± 0.35) (*p *= 0.0001). 

The CE treatment in the concentrations 1, 10 and 50 µg/mL did not present significant influence on chromosomal instability caused by hydrogen peroxide (Figure 4F) However, treatments with crude extract (100 µg/mL), ethyl acetate fraction and isolated compounds (1, 10, 50 and 100 µg/mL) reverted the chromosomal instability despite the presence of hydrogen peroxide (*p *= 0.001). Quercetin (1, 10, 50 and 100 µg/mL), rutin and isoquercitrin (50 and 100 µg/mL) and ethyl acetate (100 µg/mL) gave the best results, reversing the damage significantly compared with control group (*p *= 0.001).

### 2.5. Genotoxicity Analyzed by Alkaline Comet Assay

DNA damage induced by hydrogen peroxide exposure was evaluated using the comet assay under alkaline conditions. Table 2 shows the comet class and damage index in the lymphocyte cell cultures from crude extract, ethyl acetate fraction, quercetin, rutin, isoquercitrin and quercitrin (concentration the 50 µg/mL) obtained of the *S. buxifolia* after 1 h of exposure to H_2_O_2_. Quercetin reverted in a significant manner the damage caused by hydrogen peroxide (*p* = 0.005) when compared with control. The ethyl acetate, isoquercitrin and rutin cell cultures presented similar damage indexes between treatment groups, DNA damage was significantly decreased when compared with the hydrogen peroxide group (*p* = 0.001) and when compared to lymphocytes from the isolated quercitrin (*p* = 0.01).

**Table 2:** Evaluation of DNA damage by the comet test in leukocytes

Concentration (50 µg/mL)	Comet Class (0–4)					Index of DNA damage
	0	1	2	3	4	
Control	88 ± 3	5 ± 1	3 ± 1	2 ± 1	2 ± 1	0.12 ^a^
H_2_O_2_	42 ± 5	10 ± 2	5 ± 1	12 ± 2	29 ± 1	0.56 ^b^
Crude extract	53 ± 3	17 ± 2	11 ± 2	13 ± 3	6 ± 1	0.47 ^bc^
Ethyl acetate fraction	62 ± 6	12 ± 2	8 ± 1	14 ± 2	4 ± 1	0.38 ^c^
Quercetin	77 ± 4	13 ± 1	4 ± 1	3 ± 1	3 ± 1	0.23 ^a^
Quercitrin	46 ± 3	11 ± 2	8 ± 1	16 ± 3	20 ± 2	0.55 ^b^
Isoquercitrin	62 ± 2	10 ± 3	9 ±1	10 ± 2	9 ± 1	0.38 ^c^
Rutin	66 ± 5	16 ± 2	8 ± 1	5 ± 1	8 ± 1	0.37 ^c^

Index of DNA damage: Σ (1 + 2 + 3 + 4 comet class)/100. 0 = nucleus without DNA damage. Results are mean ± SD for n ≥ 3.

## 3. Discussion

The present investigation reported the concentration-dependent protective effects of ethyl acetate fraction, quercetin, rutin and isoquercitrin isolated from *Scutia buxifolia* against oxidative DNA damage induced by hydrogen peroxide in human lymphocytes. Crude extract and quercitrin had less effect on chromosomal instability and DNA damage.

These results are consistent with several previous studies that reported the effects of medicinal plants and their extracts rich in polyphenolic/flavonoid compounds as protective compounds against damage mediated by ROS, due to enhancement of antioxidant defenses [6,16,17]. In this study the presence of phenolic and flavonoid compounds in crude extract and ethyl acetate fraction (12.02% and 35.68%, respectively, Table 1) is directly related to their protective effects. Even low concentrations such as 1 µg/mL quercetin, rutin and isoquercitrin already provide a large effect. The *in vitro* protection against oxidative DNA damage in human lymphocytes by quercetin treatment has been described earlier [17,18,19,20]. 

Hydrogen peroxide is a reactive compound that in elevate concentrations can generate hydroxyl radicals (OH**^.^**) via the Fenton reaction. The OH has highly affinity to DNA causing strand breakage. This process can result in DNA instability, mutagenesis and ultimately carcinogenesis [18,20]. Therefore, the beneficial effects of flavonoids have been reported. The antioxidant properties include their ability to inhibit xanthine oxidase activity, to scavenge superoxide anions and hydroxyl radicals, and to inhibit lipid peroxidation as showed from a large variety of experimental systems [21]. Free radical scavenging flavonoids efficiency is associated with the presence and number of hydroxyl groups in the B-ring of the flavonoid [22].

Experimental parameters, such as growth, density and viability, can affect the cell response to a specific toxin. Hydrogen peroxide (25 µM) affected membrane integrity, the treatment with flavonoids and extracts of the *Scutia buxifolia* increased the cell viability despite the presence of peroxide hydrogen. Duthie *et al*. [17] emphasized that quercetin decreased specific oxidative DNA base damage and the cytoprotection was maintained even after the cells were washed free of flavonoid, thus avoiding its direct reaction with hydrogen peroxide, quercetin must act within the cell or cell membranes and is metabolized, possibly by the cytochrome P_450_ mixed function oxidase system [23]. In this present study, we used the comet assay, as a fast and reliable method that is able to detect genotoxicity in virtually any mammalian cell type [17,18], and only quercitrin had no protective effect in human lymphocytes exposed to hydrogen peroxide according to the comet assay.

## 4. Experimental

### 4.1. Chemicals, Apparatus and General Procedures

Methanol, hydrogen peroxide, acetic acid, gallic acid, chlorogenic acid and caffeic acid purchased from Merck (Darmstadt, Germany). Quercetin, rutin, kaempferol, trypan blue, penicillin G and streptomycin sulphate were acquired from Sigma Chemical Co. (St. Louis, MO, USA). Fetal bovine serum (FBS) and RPMI 1640 medium were purchased from GIBCO Co. (Grand Island , NY, USA). Milli-Q ultra-purified water was used in preparing the samples. High performance liquid chromatography (HPLC-DAD) was performed with a Shimadzu Prominence Auto Sampler (SIL-20A) HPLC system (Shimadzu, Kyoto, Japan), equipped with Shimadzu LC-20AT reciprocating pumps connected to a DGU 20A5 degasser with a CBM 20A integrator, SPD-M20A diode array detector (DAD) UV-VIS detector and LC solution 1.22 SP1 software (Shimadzu, Kyoto, Japan).

### 4.2. Plant Collection and Extraction

Leaves of *S. buxifolia* were collected in Dom Pedrito (Rio Grande do Sul, Brazil) in October of 2007 (coordinates 30°59′09′′ S and 54°27′44′′ W). Exsiccate was archived as voucher specimen in the herbarium of Department of Biology at Federal University of Santa Maria by register number SMBD 10919. The leaves were dried at room temperature and powdered in a knife mill (0.86 µm), resulting in a mass of 372.34 grams of plant material, which was submitted to maceration at room temperature with ethanol 70% for a week with daily shake. After filtration, the extract was evaporated under reduced pressure to remove the ethanol and after this step the aqueous extract was partitioned successively with dichloromethane, ethyl acetate and *n*-butanol (3 × 200 mL for each solvent). Detailed isolation of the flavonoids used in this experiment is published elsewhere [16].

### 4.3. Quantification of Phenolics and Flavonoids Compounds by HPLC-DAD

Reverse phase chromatographic analyses were carried out under gradient conditions using a C_18 _column (4.6 mm × 250 mm) packed with 5 μm diameter particles; the mobile phase was water containing 2% acetic acid (A) and methanol (B), and the composition gradient was: 5% (B) for 2 min; 25% (B) until 10 min; 40, 50, 60, 70 and 80% (B) every 10 min; following the method described by Laghari *et al*. [24] with slight modifications. The crude extract, ethyl acetate fraction and mobile phase were filtered through 0.45 µm membrane filter (Millipore) and then degassed by ultrasonic bath prior to use, the samples of the plant were analyzed dissolved in methanol at a concentration of 8 mg/mL. Stock solutions of standards references were prepared in methanol at a concentration range of 0.031–0.250 mg/mL for kaempferol, quercetin and rutin, and 0.006–0.250 mg/mL for gallic, chlorogenic and caffeic acids. Quantification was carried out by integration of the peaks using the external standard method, at 254 nm for gallic acid, 325 nm for caffeic and chlorogenic acids, and 365 nm for quercetin, rutin and kaempferol. The flow rate was 0.8 mL/min and the injection volume was 40 µL. Chromatographic peaks were confirmed by comparing its retention time with those of reference standards and by UV spectra (200–600 nm). Calibration curve for gallic acid: Y = 53985x + 1020.6 (r = 0.9998); chlorogenic acid: Y = 52548x + 1082.3 (r = 0.9985); caffeic acid: Y = 87846x + 1093 (r = 0.9996); rutin: Y = 103861x + 1235.8 (r = 0.9991); quercetin: Y = 150833x + 4741.7 (r =0.9999) and kaempferol: Y = 130745x + 1897.9 (r = 0.9989). All chromatography operations were carried out at ambient temperature and in triplicate. The limit of detection (LOD) and limit of quantification (LOQ) were calculated based on the standard deviation of the responses and the slope using three independent analytical curves, as defined by ICH [25]. LOD and LOQ were calculated as 3.3 and 10 σ/S, respectively, where σ is the standard deviation of the response and S is the slope of the calibration curve.

### 4.4. Treatments

To test the protective effects of *Scutia buxifolia* on cell viability and protective effect against chromosome damage in human lymphocytes we performed a protocol similar to that described by Wilms *et al*. [16]. Briefly, we preincubated the cell cultures with crude extract (CE), ethyl acetate fraction (EA) and four flavonoids (quercetin, quercitrin, isoquercitrin and rutin) isolated from *Scutia buxifolia* (1 h, 37 °C), four concentrations (1, 10, 50 100 µg/mL) of these compounds were tested here, after the cells were washed with PBS, pH 7.4, and then exposed an effective dose of hydrogen peroxide (25 µM, 1 h, 37 °C) [16]. The medium culture was used as a negative control and the medium added 25 µM hydrogen peroxide was used as positive control group.

### 4.5. Blood Collection and Lymphocyte Culture

Peripheral blood samples obtained from a female healthy volunteer (28 years of age), that did not smoke, drink, or use chronic medication was collected after 12-h overnight fasting by venipuncture using a top Vacutainer® (BD Diagnostics, Plymouth, UK). Tubes with heparin and 5 mL was used for glass to cultured lymphocytes in culture media containing 1 mL RPMI 1640 with 10% foetal calf serum (FCS) and 1% penicillin/streptomycin and phytohematoglutin (PHA). The cells were maintained in suspension culture at 37 °C in a humidified 5% CO_2_ atmosphere in RPMI 1640 growth medium (RPMI 0 medium) for 72 h. Further we exposed the cells to treatments during 1 hour with samples, and after for 1 h with hydrogen peroxide. Cell viability, comet assay, mitotic index and chromosomal instability were analyzed. All treatments were done in triplicate. 

### 4.6. Cell Viability

The cell viability was evaluated before and after treatment exposure using the Trypan blue dye exclusion method [26]. To perform the test we used 100 µL of each cell suspension, which were mixed with 100 µL of 0.4% Trypan blue solution and checked for viability 3 min later. At least 300 cells were counted for each survival determination. The cells were analyzed through microscopic observation (Neubauer chamber) and the percentage of viable cells was determined. Cell viability was expressed as a percentage of the control value.

### 4.7. Single Cell Gel Electrophoresis (Comet Assay)

The alkaline comet assay was performed as described by Singh *et al*. [27] in accordance with the general guidelines for use of the comet assay [28,29]. One hundred cells (50 cells from each of the two replicate slides) were selected and analysed. Cells were visually scored according to tail length and classified as follows: class 0 (absence of tail); class 1 (tail of up to 1× the diameter of the nucleus of negative control); class 2 (tail of up to 2× the diameter of the nucleus); class 3 (tail of up to 3× the diameter of the nucleus); class 4 (tail of more than 3× the diameter of the nucleus). Apoptotic cells were not counted [30]. Received scores from 0 (no migration) to 4 (maximal migration). Therefore, the damage index for cells ranged from 0 (all cells with no migration) to 400 (all cells with maximal migration). The slides were analysed under blind conditions by at least two different individuals.

### 4.8. Mitotic Index and Chromosomal Instability in Human Lymphocytes

After treatment exposure, one replicate of each treatment was used to investigate the mitotic index and chromosomal instability using G-band cytogenetic analysis [31]. At least 50 mitoses were analysed in each sample.

### 4.9. Statistical Analysis

All analyses were carried out using the statistical package for social studies SPSS version 12.0 (SPSS Inc., Chicago, IL). To compare the treatments in all tests we performed analysis of variance (One-way) followed by *post hoc* Tukey test. We chose these statistical tests because a previous statistical analysis performed using the Kolmogorov-Smirnov test showed normal distribution of the variables investigated here. All *p* values were two-tailed. The alpha value considered to be statistically significant was *p* = 0.001.

## 4. Conclusions

In conclusion, the crude extract, ethyl acetate fraction and flavonoids isolated obtained from *Scutia buxifolia* show protective effects against damage caused by hydrogen peroxide in human lymphocytes, possibly by decreasing oxidative stress due to their antioxidant nature [9,32]. Thus, a diet containing flavonoids could be effective in reducing baseline and exogenously induced oxidative DNA damage, however, depending on the type and amount of flavonoids, in which case the specific flavonoids supplied as with supplements are more effective.
